# The Evolution of the Meningitis Vaccine Project

**DOI:** 10.1093/cid/civ594

**Published:** 2015-11-09

**Authors:** Kathleen Tiffay, Luis Jodar, Marie-Paule Kieny, Muriel Socquet, F. Marc LaForce

**Affiliations:** 1 PATH, Ferney-Voltaire, France; 2 Pfizer, New York, New York; 3 Department of Health Systems and Innovation, World Health Organization; 4 PATH, Geneva, Switzerland; 5 Serum Institute of India, Ltd, Pune

**Keywords:** meningitis vaccine, public–private partnership, vaccine development model, project structure, public health impact

## Abstract

***Background.*** In 2001, the Meningitis Vaccine Project (MVP) was tasked to develop, test, license, and introduce a group A meningococcal (MenA) conjugate vaccine for sub-Saharan Africa. African public health officials emphasized that a vaccine price of less than US$0.50 per dose was necessary to ensure introduction and sustained use of this new vaccine.

***Methods.*** Initially, MVP envisioned partnering with a multinational vaccine manufacturer, but the target price and opportunity costs were problematic and formal negotiations ended in 2002. MVP chose to become a “virtual vaccine company,” and over the next decade managed a network of public–private and public–public partnerships for pharmaceutical development, clinical development, and regulatory submission. MVP supported the transfer of key know-how for the production of group A polysaccharide and a new conjugation method to the Serum Institute of India, Ltd, based in Pune, India. A robust staff structure supported by technical consultants and overseen by advisory groups in Europe and Africa ensured that the MenA conjugate vaccine would meet all international standards.

***Results.*** A robust project structure including a team of technical consultants and 3 advisory groups in Europe and Africa ensured that the MenA conjugate vaccine (PsA-TT, MenAfriVac) was licensed by the Drug Controller General of India and prequalified by the World Health Organization in June 2010. The vaccine was introduced in Burkina Faso, Mali, and Niger in December 2010.

***Conclusions.*** The development, through a public–private partnership, of a safe, effective, and affordable vaccine for sub-Saharan Africa, PsA-TT, offers a new paradigm for the development of vaccines specifically targeting populations in resource-poor countries.

The Meningitis Vaccine Project (MVP), a partnership between PATH and the World Health Organization (WHO), was created in 2001 through core funding from the Bill & Melinda Gates Foundation to eliminate epidemic meningitis as a public health problem in sub-Saharan Africa through the development, testing, introduction, and widespread use of conjugate meningococcal vaccines.

This project was created in response to repeated and severe group A meningococcal (MenA) meningitis epidemics in sub-Saharan Africa during the 1990s, and the potential offered by new meningococcal conjugate vaccines to prevent such epidemics. African public health leaders issued a call for action as meningitis rapidly became a public health priority given the high case fatality rates, high attack rates among young adults at the peak of their economic contributions, and the incapacitating sequelae seen in 10%–20% of survivors. Vaccination campaigns with a meningococcal C conjugate vaccine in the United Kingdom in 1999 had demonstrated that conjugate vaccines conferred herd protection within a targeted population once sufficient numbers had been vaccinated [1]. This experience demonstrated the possibility of preventing—and perhaps eliminating—MenA meningitis epidemics in the African “meningitis belt” through the development and widespread use of a MenA conjugate (PsA-TT) vaccine. Because a monovalent MenA conjugate vaccine would have limited commercial interest outside of Africa, public-sector support for vaccine development, clinical evaluation, and pilot introduction was provided through the creation of MVP [2].

Conjugate meningococcal vaccine development for Africa was initially envisaged as a partnership between MVP and a multinational “big pharma” company to produce a bivalent group A/C conjugate vaccine. In this scenario, a small MVP team at WHO and PATH would focus on managing this partnership while working to improve meningitis surveillance in Africa and planning for the vaccine's introduction in meningitis belt countries (Benin, Burkina Faso, Burundi, Cameroon, Central African Republic, Chad, Côte D'Ivoire, Democratic Republic of Congo, Ethiopia, Erithrea, Ghana, Guinea, Guinea Bissau, Kenya, Mali, Mauritania, Niger, Nigeria, Rwanda, Senegal, South Sudan, Tanzania, The Gambia, Togo, Uganda).

Yet, 18 months after the project's starting date, MVP had evolved into a “virtual company” managing a new model for public sector vaccine development that linked a network of partners with unique roles and responsibilities, and sought to transfer technology and know-how for all aspects of meningococcal conjugate vaccine production to a developing country vaccine manufacturer. This article describes the maturation of MVP's vaccine development strategy, and the project structure, guiding principles, and values that ultimately led to the introduction of a safe, highly effective, and affordable MenA conjugate vaccine for sub-Saharan Africa.

## ESTABLISHING THE VACCINE DEVELOPMENT STRATEGY

In the first year of the project, MVP embarked on 3 parallel activities that informed its vaccine development strategy: (1) needs assessment with African public health leaders; (2) extensive consultations to inform vaccine product choice; and (3) due diligence to identify vaccine development partners.

### Needs Assessment

African public health officials were key MVP partners from the start of the project. After all, it was their call to action that led to the creation of the project, and it was their countries that were impacted by the devastation of meningitis epidemics. MVP leaders sought their counsel and advice to inform decisions on vaccine development strategy through country visits and participation in the December 2001 Task Force on Immunization Meeting in Addis Ababa, Ethiopia. Interviews with public health leaders highlighted that epidemic meningitis was a major problem, that current control strategies had not worked, and that the price of any new vaccine was a key determinant of eventual impact. A WHO Regional Office for Africa (AFRO)–sponsored review on the introduction of new vaccines in Africa had identified vaccine price as the most important constraint to the introduction of new vaccines in Africa. African public health leaders asked for a safe and effective vaccine with a price of less than US$0.50 per dose.

### Product Choice

The initial plan considered 2 development options, namely, a monovalent group A or a bivalent group A/C meningococcal conjugate vaccine. Given the availability of group C meningococcal (MenC) conjugate vaccines and that group C meningitis was rare in sub-Saharan Africa and that the development costs and the complexity of a bivalent product would have been substantially higher, MVP chose to develop a monovalent group A vaccine. However, epidemiologic developments in Africa in the years 2001–2002 were characterized by a significant outbreak of group W meningitis in Burkina Faso. This event emphasized the need to consider the possibility of developing a bivalent A/W vaccine. Following technical consultations, the MVP team decided to continue with the development of a monovalent group A conjugate vaccine while increasing support for surveillance activities in West Africa. Fortunately, the group W meningitis epidemic did not spread.

Because the overarching mandate for determining MVP's vaccine product choice was public health impact (to prevent and eliminate meningococcal epidemics in the African meningitis belt), other factors that were considered in the choice of the vaccine to be developed included the following:

- Timeframe: The vaccine should be available in the shortest timeframe possible without compromising effectiveness or safety—and before the projected end of MVP in 2010.
- Safety: The vaccine must meet European Pharmacopoeia and WHO standards to obtain WHO prequalification and international licensure. Safety was a particularly important issue.
- Effectiveness: The MenA conjugate vaccine should generate robust immunity as measured by bactericidal titers, and confer herd protection as was the case with the recently introduced group C conjugate vaccines.
- Vaccine price: The vaccine should be priced at less than US$0.50 per dose.
- Single dose: A single-dose product would be highly desirable.

After extensive consultation and given that MenA caused virtually all major meningitis epidemics (defined as an incidence rate >100/100 000) and accounted for about 85% of all meningitis cases in sub-Saharan Africa [3, 4], MVP decided to focus on developing a single-dose monovalent MenA conjugate vaccine for use in 1- to 29-year-olds.

For sustainable public health impact, this vaccination strategy would need to be complemented by an infant strategy following initial vaccine rollout. For this reason, following licensure for the target age group, MVP continued with infant studies with the MenA conjugate vaccine to identify ideal schedule and dosage for ultimately integrating the vaccine into Expanded Programme on Immunization schedules in targeted countries. The infant strategy was approved by the WHO Strategic Advisory Group of Experts on Immunization in October 2014.

### Negotiations With Multinational Pharmaceutical Companies

MVP leadership initially assumed that since most of the MenA conjugate vaccine development and production know-how was in the private sector, MVP funds would be transferred to a single vaccine manufacturer as a push mechanism to support research and development and clinical trials, and that the vaccine manufacturer would commercialize the vaccine at an agreed price and quantity. During 2001, MVP received proposals from 2 multinational vaccine manufacturers that required major upfront investments to develop and manufacture a vaccine with a price of about US$2.00 per dose, with the per-dose cost increasing significantly if <25 million doses were purchased annually. In subsequent negotiations, MVP emphasized the need for a lower price point based on the needs assessment with African countries. Companies concluded that the combination of low price and opportunity costs that would compete with other projects in their research pipeline made the MVP collaboration unacceptable, and by mid-2002 it was clear that MVP would be unable to successfully conclude negotiations with a “big pharma” company.

Although initially discouraging, the failure to close an agreement with a pharma company created a remarkable opportunity for MVP. With the motivation of the urgent public health need, a fully funded 10-year project, and support from world-class scientific and technical experts, MVP was able to pursue an alternative strategy responsive to its public health mandate.

### A New Vaccine Development Model

In the first half of 2002, MVP convened a series of meetings with WHO, PATH, and experienced industry consultants to evaluate alternative vaccine development options. To facilitate the work, project leaders identified critical components:

- The availability of affordable intellectual property for a conjugation method
- Companies able to provide the raw materials (group A polysaccharide [PsA] and tetanus toxoid [TT]) needed to make the vaccine
- The feasibility of technology transfer of the conjugation method to a vaccine manufacturer likely based in a developing country
- Scale-up of the conjugation process to industrial scale at the developing country vaccine manufacturer
- Conduct of the necessary clinical studies to ensure the safety, immunogenicity, and efficacy of the vaccine
- Regulatory approvals for the new vaccine
- Vaccine introduction

**Figure 1.**
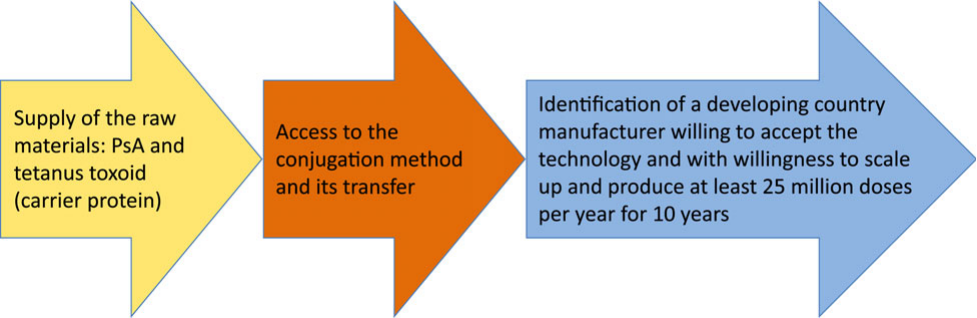
Design of pharmaceutical development. Source: Meningitis Vaccine Project. Abbreviation: PsA, group A polysaccharide.

MVP concluded that it was possible to develop a MenA conjugate vaccine that would cost less than US$0.50 per dose as long as the annual production was >25 million doses. The design of the pharmaceutical development of the vaccine had 3 principal elements (Figure 1).

### Demonstrating Feasibility and Commitment to High Standards

After announcing its intention to move forward with this unconventional vaccine development approach, MVP faced increasing scrutiny from the global health community about the feasibility of the approach and fears that the possibility of working with a developing country vaccine manufacturer would result in a lower-quality vaccine. This latter concern was particularly sensitive as meningitis belt countries are some of the most impoverished countries in the world, leading to concerns that MVP would be developing “a poor vaccine for the poor.”

To respond to these concerns, MVP invested significant resources in due diligence and consultations while compiling detailed plans and information to help convince stakeholders of the viability of the proposed vaccine development model [2]. Ten international meetings in Africa, Europe, and the United States were held during 2002 that included African ministers of health, WHO AFRO, the MVP Management Committee, PATH's Strategic Advisory Committee, the Bill & Melinda Gates Foundation, WHO's Global Vaccine Forum, WHO Strategic Advisory Group of Experts on Immunization (SAGE), the WHO Consultative Meeting on Prevention and Control of Epidemic Meningococcal Meningitis, and the MVP scientific Expert Panel.

#### Establishing High Standards for the MenA Conjugate Vaccine

To address quality concerns, the MVP team committed to meeting European Medicines Agency standards and WHO safety standards for the MenA conjugate vaccine in addition to all national regulatory authority requirements in the country where the vaccine would be produced. Thus, MenA conjugate vaccine development would proceed according to established international standards: Good Manufacturing Practices, Good Laboratory Practices, and Good Clinical Practices.

Product development plans, all stages of the vaccine development, and critical vaccine development decisions would receive detailed analysis and approval from an MVP Expert Panel comprised of international scientific experts in conjugate vaccines, epidemiology, public health, vaccine pharmaceutical and clinical development, and vaccine regulatory processes. MVP's Management Committee and the African Project Advisory Group would provide additional oversight to MVP vaccine development.

With rigorous adherence to these commitments, MVP was able to effectively address concerns about vaccine safety and efficacy with an alternative model for vaccine development.

#### Selection of Key Vaccine Development Partners

Identification of the 3 key pharmaceutical partners with the desired skills, capacity, and interest who would commit to a collaboration with MVP was a critical task for the small MVP team and its expert pharmaceutical consultants during 2002.

The first confirmed partner was SynCo Bio Partners in the Netherlands, who agreed to supply PsA, the major vaccine component, and to establish seed banks and a “turnkey” process to transfer fermentation and purification processes to the manufacturing partner.

The Serum Institute of India, Ltd (SIIL), a respected vaccine manufacturer already producing and selling several WHO-prequalified vaccines, was chosen as MVP's manufacturing partner. This selection was based on extensive due diligence of 5 global manufacturers, each having at least 1 WHO-prequalified vaccine. The evaluation process required multiple consultations with experts and advisory groups. SIIL had a strong performance record for meeting timelines, a robust management structure, financial health, excellent physical facilities, a skilled technical team, and a strong interest in MVP's public health vision and guiding principles. SIIL agreed to develop a MenA conjugate vaccine at less than US$0.50 per dose, and to provide the necessary TT component of the vaccine.

**Figure 2.**
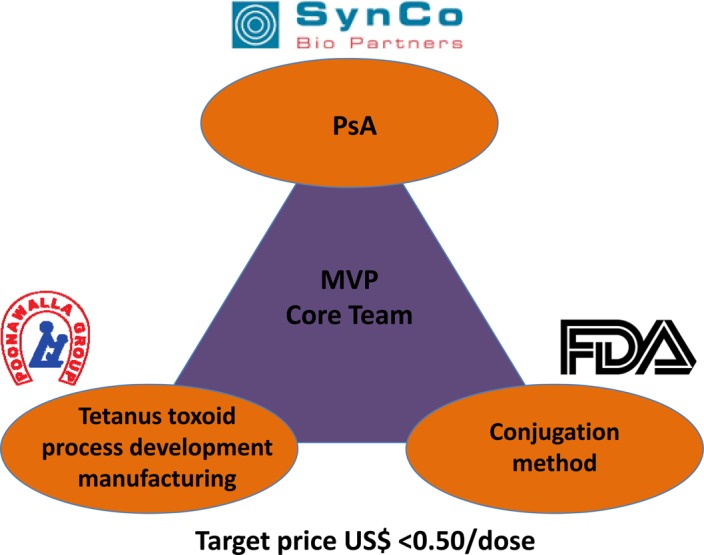
Endorsement of the MVP vaccine development consortium.

Source: Meningitis Vaccine Project. Abbreviations: CBER/FDA, Center for Biologics Evaluation and Research/US Food and Drug Administration; MVP, Meningitis Vaccine Project; Ps, polysaccharide; PsA, group A polysaccharide.

MVP faced its most daunting challenge in identifying appropriate and affordable intellectual property for a conjugation methodology. A European biotech company was initially selected to provide the conjugation technology and worked with MVP for almost a year before withdrawing from the project. MVP immediately restarted due diligence to identify either a replacement conjugation method or an alternative vaccine development partnership and contacted 8 institutions and companies, 4 of which submitted proposals to MVP. Following site visits and technical consultations, these groups presented their proposals to a meeting of the MVP Expert Panel that had been expanded to include members from SAGE. In October 2003, MVP established a new partnership with the Center for Biologics Evaluation and Research (CBER) of the US Food and Drug Administration (FDA). CBER/FDA agreed to sublicense a conjugation method at a negligible cost, and to support technology transfer of this method to SIIL. MVP stakeholders and advisors endorsed the MVP vaccine development consortium (Figure 2).

During 2002, MVP also completed competitive processes to identify laboratories for analytic and serologic work that would be a core element of the clinical studies. The National Institute for Biological Standards and Control (NIBSC) in the United Kingdom had played a critical role in the licensure of the MenC conjugate vaccine and agreed to study all specimens that were part of the development process for the new MenA conjugate vaccine. MVP then concluded contractual arrangements with the US Centers for Disease Control and Prevention for all enzyme-linked immunosorbent assay studies, and with the UK Public Health Laboratory Services for the serum bactericidal assays.

As MVP was expanding its network of vaccine development partners, it became clear that MVP was now a “virtual pharmaceutical company,” which required a substantial increase in personnel to meet management, technical, administrative, and contracting demands.

## MENINGITIS VACCINE PROJECT STRUCTURE

The MVP structure was developed to accommodate the core team across PATH and WHO, 3 advisory groups, and multiple partners and consultants, as well as having the capacity to respond to changing project needs throughout the life of the project.

The foundation of the MVP structure is the PATH/WHO partnership. Each organization brought different and complementary strengths to the project, and by defining clear roles and responsibilities, a solid partnership was established. PATH's capabilities in product development, partnership with private sector, and project management, finance, and administration, along with WHO's knowledge of vaccine introduction, strong presence in meningitis belt countries, and expertise in disease surveillance, evolved into a strong working group. However, the distinct overall organizational cultures could have undermined the project without the careful attention paid by MVP leadership to creating a strong shared vision and team identity as MVP rather than PATH or WHO. Team-building efforts focused on understanding each organization's unique characteristics to avoid frustrations and instead to take advantage of these differences. The scope, durability, and complexity of the WHO/PATH MVP partnership is testimony to the seriousness that both institutions granted to the mission. The success of MVP provided a model for subsequent projects.

### Building the MVP Team for an Expanded Vaccine Development Role

As a virtual pharmaceutical company, MVP would be reliant on a network of partners including consultants, private-sector companies, and public-sector organizations to implement the different elements of the vaccine development process. The success of the project would largely depend on a shared vision and mutual trust, communication, monitoring, and rapid problem resolution. Financial management requirements also increased significantly with the new vaccine development model, and there were increased responsibilities for ongoing interactive communication with partners, advisory groups, countries, and the global health community.

The MVP team would need technical capacities to meet immediate needs, and the flexibility to meet shifting technical and scientific requirements. MVP requirements for expertise over the life of the project included epidemiology, surveillance, intellectual property, licensing agreements, pharmaceutical development, clinical development, regulatory pathways and requirements, vaccine manufacturing, and vaccine introduction. Determining which of these capabilities to include on the core team, and which to outsource, or when to combine the 2 capabilities, were questions that MVP faced when establishing its initial team.

By the second half of 2002, MVP was expanding its staff beyond the original 4-person team (MVP Director, MVP Senior Administrator, MVP WHO Focal Point, and MVP WHO Administrative Assistant), and by early 2003, 11 additional team members with a broad range of required expertise were based at WHO Geneva, the PATH office in Ferney-Voltaire, France, and the WHO Inter-country Support Team office in Ouagadougou, Burkina Faso. While defining and recruiting full-time staff, MVP required support from a dedicated group of consultants and advisors that included global experts in vaccine development (3 vaccine development consultants for MVP—Costante Ceccarini, Jean Petre, and Neil Ravenscroft—were essential to the project's success and functioned as part of the MVP team), a life sciences transactional attorney, and business development consultants with expertise in microbiology, intellectual property, and licensing. MVP embraced these individuals as full team members and they collectively responded with enthusiasm for and commitment to the project.

### MVP Governance, Advisory Groups, and Accountability

Critical to the MVP's success was an effective governance and advisory structure comprised of the Project Management Committee (PMC), the Project Advisory Group (PAG), and a scientific Expert Panel who provided advice and support to project decisions and implementation. Through these groups, project partnerships, and formal and informal consultations across multiple public health committees and international groups, MVP cultivated a circle of talented professionals whose interest in the success of the project has been a crucial asset.

#### Project Management Committee

The PMC, established at the onset of the project, was composed of 2 senior leaders from both PATH and WHO, and functioned as the “MVP Board.” Members met in December and June of each year to review and to approve the annual workplan and budget and to monitor project progress. The PMC also advised on strategic decisions related to product development and vaccine introduction through their participation in other MVP advisory groups.

#### Project Advisory Group

The PAG was created in 2002 as a mechanism to closely involve African public health experts in the life of the project and to ensure a consensus on major decisions. It was set up as an official WHO advisory group that reported to the WHO AFRO Director of Disease Control. The PAG reviewed project progress and participated in resolution of strategic and, in particular, “political” challenges faced during project implementation. As the project neared vaccine introduction, their role expanded as members undertook advocacy roles and mobilized political commitment and resources at the regional and country level.

#### MVP Expert Panel

The Expert Panel, established in 2002, was composed of African, European, and North American vaccine development experts, and in 2003 the membership was expanded to include 2 SAGE members. This group provided technical advice and input at all critical decision points for vaccine research and development, and contributed to the growing credibility of the MVP approach.

### Partnerships

The MVP network of vaccine development partners rapidly expanded from the 3 primary pharmaceutical partners to a complex consortium that included multiple clinical sites, clinical research organizations, toxicology and serology laboratories, and regulatory experts, coordinated and supported by the project team.

MVP succeeded in bringing together people and partners who shared a common commitment to public health, which helped generate a solid context for the trust that characterized the myriad MVP partnerships throughout the life of the project. Other factors that characterized MVP partnerships were excellent partner agreements and contracts and a commitment to capacity building and strong technical support.

#### Building and Maintaining Trust in the MVP Collaboration

Trust within the MVP consortium was as important to MVP success as the science, and MVP devoted a significant amount of time and energy to establishing and fostering trust. When selecting a partner, MVP ensured that the partner was not only technically competent but was committed to the goal of the project. The project team then maintained ongoing communication on project status, issues, and upcoming objectives including regular face-to-face meetings at partner sites to clarify roles and responsibilities and to discuss and resolve issues. With partners based in countries around the world, MVP adapted work processes to each partner's culture and habits.

MVP viewed the contracting process as an important opportunity not only to generate useful agreements but also to build relationships and to promote a shared vision around the broader goals of the project. A Cooperative Research and Development Agreement was negotiated with CBER/FDA for technology transfer of their conjugation technology, and ongoing scale-up support from laboratory scale to pilot scale. A license agreement for the CBER conjugation technology was concluded with the US National Institutes of Health granting a nonexclusive license covering 2 patents related to conjugate meningococcal vaccines.

A long-term sublicensing and supply agreement was negotiated with SIIL that provided for the sublicense of the conjugation technology to SIIL, as well as for the manufacture and supply of approximately 250 million doses of the MenA conjugate vaccine using this technology.

As part of the agreement, MVP contributed toward the purchase of industrial equipment to enable the development and production of initial clinical batches of the MenA conjugate vaccine in an existing SIIL facility. SIIL subsequently decided to design, build, and validate a new building dedicated to the production of 2 conjugate vaccines—MenAfriVac and *Haemophilus influenzae* type b conjugate – to ensure sufficient production capacity to deliver the agreed 25 million doses of MenAfriVac per year. The SIIL/PATH sublicensing and supply agreement required lengthy due diligence and research in pricing and price modeling, patent reviews, assessments of product liability, and review of the relevant legal and policy environments in India and Africa. The final terms negotiated in the commercial agreement with SIIL were particularly important as they guaranteed priority for initial supplies of the vaccine to meningitis belt countries, and outlined cost parameters for public sector sales at US$0.40 cents per dose.

As the project evolved and milestones were met, trust between partners became more robust but still needed to be carefully monitored. Both internally and publicly, MVP worked to recognize the critical role of all partners in project accomplishments.

#### Commitment to Capacity Building and Technical Support to Partners

An MVP guiding principle specified that “the project is about public health impact and not simply making vaccines available.” MVP aspired to leave behind increased knowledge, experience, and capacity among its network of partners in addition to achieving the project goal of eliminating epidemic meningitis as a public health problem in sub-Saharan Africa.

Therefore, and whenever possible, MVP selected partners based in Africa and India, and capacity building was sometimes required to ensure that partners had the requisite skills and knowledge to complete project activities in compliance with national and international regulatory requirements. The MVP team and its consultants provided continuous support to partners and were constantly available to help resolve technical, scientific, and sometimes human problems. MVP technical support included not only specific training, but also assessment visits, and the provision of documents and templates when necessary. This work was further refined through comprehensive and frequent monitoring by teleconferences, technical review meetings, and mock inspections.

As the main vaccine development partner for the project, SIIL benefited from capacity building by the project team and consultants for all phases of vaccine development, including the previously described technology transfers from SynCo Bio Partners and CBER/FDA. MVP provided access to technical support in fermentation, conjugation, formulation, manufacturing, production capacity, and quality systems. The SIIL clinical team worked closely with MVP for clinical study design, implementation, and monitoring of clinical studies, and for the preparation of a regulatory file meeting the highest international regulatory requirements to obtain vaccine licensure.

Other groups whose skills and knowledge were strengthened by MVP investment in capacity building include the following:

- Indian regulatory authorities were trained in developing laboratory assays, analyzing clinical samples, and assessing regulatory information related to development of conjugate vaccines by NIBSC, FDA, and MVP staff.
- National regulatory authorities in several meningitis belt countries were trained by WHO Quality, Safety, and Standards team and MVP staff.
- Eight clinical study sites in Africa and India were provided with standard operating procedures and trained by MVP staff and consultants on all aspects of conducting clinical studies at the highest international regulatory standards. Financial and administrative support was also provided when necessary.
- Indian and African Clinical Research Organizations (CROs) worked with MVP staff to develop monitoring strategies for the clinical trials.
- Meningitis belt country laboratories gained capacity in advanced testing methods for detecting infecting organisms.
- Meningitis belt country surveillance teams benefited from MVP investments in surveillance and data collection training.

The investments in capacity building and technical support not only resulted in sustainable public health impact, but also returned benefits to the project as the vaccine was expeditiously licensed. Several clinical sites were able to support additional vaccine trials, CROs became more autonomous as the project evolved, and the improved surveillance data yielded sound information before and after the introduction of the MenA conjugate vaccine—commercialized as MenAfriVac.

## MVP SUCCESS FACTORS

A new MenA conjugate vaccine was developed, tested, and introduced within the project's 10-year timeframe and budget through an innovative vaccine development strategy through a public–private partnership that focused on making a high-quality and affordable vaccine for meningitis belt countries.

There were many factors that drove the success of MVP's vaccine development strategy, and a few are highlighted as follows:

- Public health impact was the key criterion for critical project decisions such as the vaccine development strategy.
- African countries and public health leaders were priority project “customers” as well as engaged partners.
- A strong PATH/WHO partnership evolved over time that emphasized the added value that each organization could bring to the project.
- Excellent commercialization agreements and contracts facilitated management by clarifying responsibilities and accountability.
- Progressive ownership of MVP was experienced by African governments, health systems, and communities, as well as WHO, UNICEF, Gavi, and public- and private-sector partners.
- Solid upfront project funding allowed for judicious but important risk-taking, particularly in the early years of the project.
- Global scientific and technical expertise ensured that the MenA conjugate vaccine met all international safety and efficacy standards.
- The project included a motivated, industrious, capable, and experienced core team at PATH and WHO along with their consultants.

With the current shift in public health projects to shorter funding periods and incremental funding based on frequent detailed progress reports, MVP's story is an important reminder that major public health projects, and especially those that involve innovation, rely on sufficient funds over a longer timeframe plus donor tolerance of risks that are part of innovation. At several critical moments, MVP had to regroup and discover a different direction or option for the project. Importantly, these detours ultimately added value to the project and contributed to MVP achievements and impact. MVP success is concrete evidence that an overarching public health goal combined with good science and technical expertise, a robust and inclusive structure, sound management, and a shared vision and guiding principles are a powerful combination for realizing public health impact.

## References

[1] Maiden MC, Ibara-Pavón AB, Urwin R et al Impact of meningococcal serogroup C conjugate vaccines on carriage and herd immunity. J Infect Dis 2008; 197:237–43. 10.1086/527401 PMC6767871 18271745

[2] Jodar L, LaForce FM, Ceccarini C, Aguado MT, Granoff D Meningococcal conjugate vaccine for Africa: a model for development of new vaccines for the poorest countries. Lancet 2003; 361:1902–4. 12788589 10.1016/S0140-6736(03)13494-0

[3] Traoré Y, Njanpop-Lafourcade BM, Adjogble KLS et al The rise and fall of epidemic *Neisseria meningitidis* serogroup W135 meningitis in Burkina Faso, 2002–2005. Clin Infect Dis 2006; 43:817–22. 16941360 10.1086/507339

[4] Campagne G, Schuchat A, Djibo S, Ousséini A, Cissé L, Chippaux JP. Epidemiology of bacterial meningitis in Niamey, Niger, 1981–96. Bull World Health Org 1999; 77 (6):499–508. 10427935 PMC2557688

